# Bioactive ZnO Nanoparticles: Biosynthesis, Characterization and Potential Antimicrobial Applications

**DOI:** 10.3390/pharmaceutics15112634

**Published:** 2023-11-16

**Authors:** Md. Amdadul Huq, Md. Aminul Islam Apu, Md. Ashrafudoulla, Md. Mizanur Rahman, Md. Anowar Khasru Parvez, Sri Renukadevi Balusamy, Shahina Akter, Md. Shahedur Rahman

**Affiliations:** 1 Department of Food and Nutrition, College of Biotechnology and Natural Resource, Chung-Ang University, Anseong 17546, Republic of Korea; 2 Department of Nutrition and Hospitality Management, The University of Mississippi, Oxford, MS 38677, USA; aminul.btge@gmail.com; 3 Department of Food Science and Technology, Chung-Ang University, Anseong 17546, Republic of Korea; ashrafmiu584@gmail.com; 4 Department of Biotechnology and Genetic Engineering, Faculty of Biological Science, Islamic University, Kushtia 7003, Bangladesh; mmrahmanbtg79@hotmail.com; 5 Department of Microbiology, Jahangirnagar University, Savar, Dhaka 1342, Bangladesh; khasru73@juniv.edu; 6 Department of Food Science and Technology, Sejong University, Seoul 05006, Republic of Korea; renucoimbatore@gmail.com; 7 Department of Food Science and Biotechnology, Gachon University, Seongnam 13120, Republic of Korea; shahinabristy16@gmail.com; 8 Department of Genetic Engineering and Biotechnology, Jashore University of Science and Technology, Jashore 7408, Bangladesh

**Keywords:** ZnONPs, biosynthesis, characterization, antimicrobial applications, antimicrobial mechanisms

## Abstract

In recent years, biosynthesized zinc oxide nanoparticles (ZnONPs) have gained tremendous attention because of their safe and non-toxic nature and distinctive biomedical applications. A diverse range of microbes (bacteria, fungi and yeast) and various parts (leaf, root, fruit, flower, peel, stem, etc.) of plants have been exploited for the facile, rapid, cost-effective and non-toxic synthesis of ZnONPs. Plant extracts, microbial biomass or culture supernatant contain various biomolecules including enzymes, amino acids, proteins, vitamins, alkaloids, flavonoids, etc., which serve as reducing, capping and stabilizing agents during the biosynthesis of ZnONPs. The biosynthesized ZnONPs are generally characterized using UV-VIS spectroscopy, TEM, SEM, EDX, XRD, FTIR, etc. Antibiotic resistance is a serious problem for global public health. Due to mutation, shifting environmental circumstances and excessive drug use, the number of multidrug-resistant pathogenic microbes is continuously rising. To solve this issue, novel, safe and effective antimicrobial agents are needed urgently. Biosynthesized ZnONPs could be novel and effective antimicrobial agents because of their safe and non-toxic nature and powerful antimicrobial characteristics. It is proven that biosynthesized ZnONPs have strong antimicrobial activity against various pathogenic microorganisms including multidrug-resistant bacteria. The possible antimicrobial mechanisms of ZnONPs are the generation of reactive oxygen species, physical interactions, disruption of the cell walls and cell membranes, damage to DNA, enzyme inactivation, protein denaturation, ribosomal destabilization and mitochondrial dysfunction. In this review, the biosynthesis of ZnONPs using microbes and plants and their characterization have been reviewed comprehensively. Also, the antimicrobial applications and mechanisms of biosynthesized ZnONPs against various pathogenic microorganisms have been highlighted.

## 1. Introduction

Nanoparticles (NPs) have been proposed as an intervention approach for suppressing microbial growth, as well as contamination, because of their high surface-to-volume ratio. They have distinctive chemical and physical properties that may interfere with bacterial adaptation [1]. Due to their numerous uses in disciplines of research like the health sector, agriculture, textiles, food technology, electronics, and so on, nanoparticles have attracted the attention of scientists [2,3,4,5,6]. However, due to their high propensity, NPs can survive in the environment, and this persistent attribute may be a viable tactic for preventing the bacterial growth used in the manufacturing of various food products including meat products, dairy or vegetables products, sausage products, etc. [7].

Numerous nanomaterials, such as zinc oxide nanoparticles (ZnONPs), silver nanoparticles (AgNPs), gold nanoparticles (AuNPs) and titanium dioxide nanoparticles (TiO_2_NPs), have potent capacity to both fight bacteria and prevent microbial adhesion, as well as contamination [6,7,8,9,10,11]. Among them, ZnONPs have received a lot of attention due to their safe and non-toxic nature and powerful antibacterial characteristics, which are related to the release of reactive oxygen species (ROS) on their surface [12,13,14,15]. ZnONPs outperform their bulkier counterparts in terms of antibacterial activity because of quantum confinement and size effects [16]. Due to the multiple ways ZnONPs prevent bacterial development, they can succeed easily to protect bacterial-contamination-associated diseases in humans, whereas conventional antibiotics face difficulties to prevent the development of bacterial resistance [12]. Due to its benign properties, ZnO has “generally recognized as safe” (GRAS) classification, and the antimicrobial efficacy of ZnONPs indicates that they are a potent antimicrobial agent for preventing foodborne pathogen contamination in the food sector [17].

These nanoparticles are often created via physical and chemical processes like photochemistry, chemical reduction and microwave irradiation [18,19,20]. The main problems of these techniques are that they are costly, involve labor-intensive equipment and produce harmful consequences due to the use of poisonous substances [21,22]. Nowadays, scholars are focusing on biological strategies for affordable and simple production of nanoparticles due to the different limitations of physicochemical methods. Biological synthesis is a facile, rapid, cost effective, non-toxic and ecofriendly productive method because it is not very expensive, and it can also substitute toxic chemicals and decrease capping and stabilizing agents. A variety of biological resources such as plants and their various parts and different microbes (bacteria, algae, fungi, etc.) could be used for the facile and green synthesis of bioactive nanoparticles [23,24,25,26,27].

A serious problem for global public health is antibiotics resistance. Antibiotics resistance is mostly a result of the abuse of antibiotics. The overuse of antibiotics to treat bacterial infections in humans and aquatic animals has resulted in the spread of numerous antibiotic-resistant strains into the environment [28,29,30]. Since numerous infectious diseases that might be fatal are brought on by pathogenic bacteria, multidrug-resistant microorganisms pose a severe threat to public health globally [31,32,33]. Due to mutation, shifting environmental circumstances and high drug use, the number of multidrug-resistant bacterial strains is continuously rising. To solve this issue, researchers are working to create novel medications for the treatment of these microbial illnesses [9,34]. Biosynthesized ZnONPs could be novel and effective agents to control these multidrug-resistant pathogenic microorganisms because of their safe and non-toxic nature and powerful antibacterial characteristics. Many recent studies have shown that different pathogenic microorganisms can be successfully controlled using biosynthesized ZnONPs [15,35,36,37,38]. This review emphasizes the facile and rapid biological synthesis of ZnONPs using both microbes and plants and their characterizations, potential antimicrobial applications and antimicrobial mechanisms against pathogenic microorganisms.

## 2. Biosynthesis of ZnONPs

**Figure 1:**
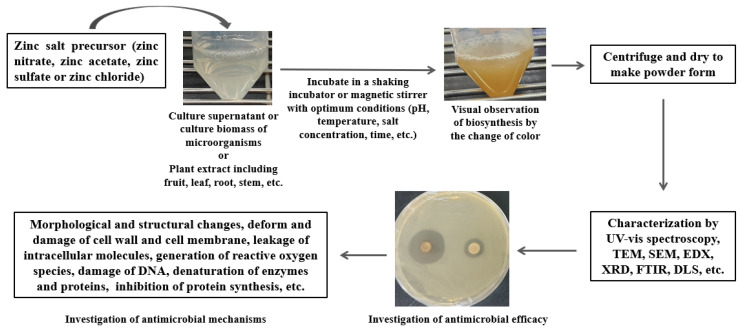
Schematic illustration of biosynthesis and potential antimicrobial applications of bioactive ZnONPs.

Biosynthesis of ZnONPs is a simple, facile, cost-effective and eco-friendly method compared to the physical and chemical methods that produce various toxic by-products that could be dangerous for our environment [39]. Moreover, biosynthesized nanoparticles are more biocompatible and show significantly higher antimicrobial activity than chemically or physically synthesized nanoparticles [40,41]. For these reasons, scientists are focusing more on utilizing different biological resources for the green, safe and effective synthesis of ZnONPs [3,8,23,36]. For the biosynthesis of ZnONPs, different microorganisms such as bacteria, fungi, yeast or various parts of plants such as leaf, root, fruit, flower, peel, stem, etc. could be used. Figure 1 shows the various steps of facile, cost-effective and eco-friendly biosynthesis of bioactive ZnONPs using the extracts of plants and microbes and their potential antimicrobial efficacy against pathogenic microorganisms.

### 2.1. Microbe-Mediated Biosynthesis of ZnONPs

Microbe-mediated nanoparticles (NPs) have recently received a lot of attention because of the availability of microorganisms, their easy reproduction and their safe utilization for the biosynthesis of nanoparticles [27,39,42]. Chemical and physical methods can be used to produce NPs, but the microbial synthesis of ZnONPs is considerably more useful than other methods due to their environmental friendliness and low cost. Because of their prevalence in living microorganisms, ZnONPs have become quite popular among other nanoparticles. ZnONPs can be produced by microbial cells, proteins and a variety of enzymes in both prokaryotes and eukaryotes [43]. There are many recent studies on the cost-effective biosynthesis of ZnONPs using various microorganisms such as bacteria, fungi, yeast, algae, etc. (Table 1). Both intracellular and extracellular methods can be used for the facile and eco-friendly synthesis of ZnONPs using microbes [8,13,44]. The culture supernatant of microorganisms and the microbial biomass contain different bioactive compounds including enzymes, proteins, amino acids and many other biomolecules that serve as reducing, capping and stabilizing agents during the synthesis process [30,39]. Previous studies have reported that the bioreduction of Zn^2+^ was initiated by the electron transfer from NADH by an NADH dependent reductase enzyme that acts as an electron carrier. Consequently, the ZnONPs are formed. Subsequently, various biomolecules such as proteins, amino acids, flavonoids, etc. attached with ZnO and stabilized the ZnONPs [39]. It is also reported that the amino acids present in the proteins were found to interact with the Zn^2+^ ions to form ZnONPs [39].

**Figure 2:**
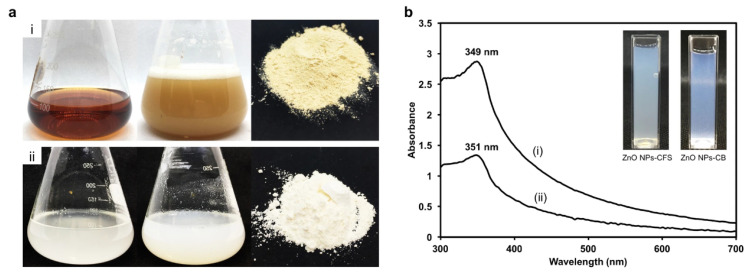
(**a**) Reduction of Zn^2+^ to ZnONPs by (i) cell-free supernatant and (ii) cell biomass of *L. plantarum* TA4. (**b**) UV-Vis spectrum of (i) ZnONPs-CFS and (ii) ZnONPs-CB. This figure has been reprinted with permission from Ref. [13], copyright 2020, Nature Portfolio.

Yusof et al. [13] reported the *Lactobacillus plantarum* TA4 mediated the biosynthesis of ZnONPs using both intracellular and extracellular methods (Figure 2). They added the zinc nitrate solution to the cell-free supernatant (CFS) for the extracellular biosynthesis of ZnONPs; as well, the cell biomass (CB) was added to the zinc nitrate solution for the intracellular biosynthesis of the ZnONPs. The synthesis of NPs was confirmed by visual observation. The synthesized ZnONPs were collected by high-speed centrifugation and dried at 100 °C to obtain the powder form. Through the FTIR analysis, they found different biomolecules present both in the cell-free culture supernatant and in the cell biomass, as well as in the synthesized ZnONPs, and concluded that these biomolecules may be involved as reducing and capping agents during the biosynthesis process [13]. 

Kumar et al., 2022 [3] reported the extracellular synthesis of bioactive ZnONPs using fungal isolate (*Aspergillus* sp.). The authors added the culture supernatant dropwise into the zinc acetate solution and confirmed the biosynthesis of ZnONPs by visual observation of color change [3]. Abdo et al. [44] successfully synthesized ZnONPs using cell-free filtrate of *P. aeruginosa*. They concluded that various metabolites present in the cell-free culture supernatant of *P. aeruginosa* are responsible for the formation and stabilization of the synthesized ZnONPs [44]. Suba et al. [8] demonstrated the intracellular biosynthesis of ZnONPs using the cell biomass of *Lactobacillus* spp. within 24 h of reaction and found spherical-shaped ZnONPs with a 32 nm average size. Abdelhakim et al. [24] used endophytic fungi *Alternaria tenuissima* for the extracellular production of spherical-shaped ZnONPs that possess significant antimicrobial activity against different pathogenic microbes. Table 1 summarizes the microbe-mediated biosynthesis of ZnONPs and their potential antimicrobial applications.

### 2.2. Plant-Mediated Biosynthesis of ZnONPs

Plant-extract-mediated biosynthesis of ZnONPs has been revealed as a viable option due to its convenience, stability and ease of synthesis compared to all other organisms. Mostly during the synthesis of ZnONPs, extracted phytochemicals function as reducing and capping agents. In the synthesis of ZnONPs as a natural green medium for metallic ion reduction, active bioorganic chemicals in plant extract were crucial [60]. Plant-based NP production has various advantages including minimal cost, ease of use, fast production time, reliability and the ability to scale up production volumes [61]. Furthermore, the availability of bioorganics with many active chemicals in plant components increases demand for ZnONPs, resulting in low-cost, secure and simple syntheses [60]. Various parts of plants such as the roots, shoots, fruits, seeds, leaves, etc. were utilized for the rapid, facile and eco-friendly synthesis of ZnONPs. There are many recent reports on the biosynthesis of ZnONPs and their potential antimicrobial applications using different parts of plants (Table 2). Abomuti et al. [62] reported the plant-mediated biosynthesis of bioactive ZnONPs using the leaf extract of *Salvia officinalis*. They added aqueous leaf extract to the zinc nitrate solution under constant stirring at a 50 °C temperature. In the second step, they added NaOH solution dropwise under continuous stirring at 50 °C to maintain the stable pH of the reaction mixture. Finally, the biosynthesized ZnONPs were collected by centrifugation and dried to obtain the powder form. Through the FTIR analysis, they found different biomolecules including phenolic and flavonoid compounds present in both the aqueous leaf extract of *Salvia officinalis* and the synthesized ZnONPs and concluded that these biomolecules may be involved as reducing and capping agents during the biosynthesis process [62]. Fouda et al. [63] reported that the peel extract of *Punica granatum* mediated the synthesis of bioactive ZnONPs. The author added the aqueous peel extract of *Punica granatum* into the zinc acetate solution and confirmed the biosynthesis of ZnONPs by visual observation of color change [63]. Fruit extract of *Myrica esculenta* was used by Lal et al. [23] for the rapid and eco-friendly synthesis of ZnONPs. Urge et al., 2023 [2] successfully synthesized ZnONPs using the bulb extract of *Allium sativum* and the root extract of *Zingiber officinale*. They identified various functional groups associated with the formation of ZnONPs [2]. Alotaibi et al. (2022) [64] demonstrated the biosynthesis of ZnONPs using the leaf extract of *Gardenia thailandica* within 1 h of reaction and found spherical-shaped ZnONPs with a 37.4 nm average size. The leaf extract of *Carica papaya* was used for rapid and green synthesis of bioactive ZnONPs [65]. Menazea et al. (2021) [66] used the peel extract of orange for the rapid and facile synthesis of cubic-shaped ZnONPs. Suručić et al. (2020) [67] synthesized ZnONPs using flower extract of *Geranium robertianum*. Plant extract contains various biomolecules such as enzymes, proteins, amino acids, flavonoids, terpenoids and phenolic compounds that play significant roles during the biosynthesis of ZnONPs as reducing and capping agents. Table 2 summarizes the plant-mediated biosynthesis of ZnONPs and their potential antimicrobial applications.

## 3. Critical Parameters for Rapid and Stable Biosynthesis of ZnONPs

Different parameters significantly affect the rapid and stable synthesis of ZnONPs. Several critical parameters have been identified for the rapid and stable synthesis of ZnONPs, including the concentration of the plant extract and metal salt, incubation time, temperature, pH and stirring rate. The optimal conditions for each parameter may vary depending on the specific plant extract or microbial species and metal salt used. However, some general trends have been observed, such as higher concentrations of plant extracts and metal salts leading to larger yields of nanoparticles, longer incubation times leading to larger particle sizes and higher temperatures leading to faster reaction rates [86,87,88]. The pH of the reaction also significantly affects the rate of ZnONP formation [62,89].

### 3.1. Factors Influencing the Mass Production of ZnONPs

The mass production of ZnONPs can be affected by several parameters, including the concentration of plant extracts and metal salts, incubation time, temperature, and pH. Higher concentrations of plant extracts and metal salts generally lead to larger yields of nanoparticles, although there may be an optimal concentration beyond which further increases have little effect. Longer incubation times generally lead to larger particle sizes, which can affect the stability and biocompatibility of the nanoparticles. Higher temperatures can lead to faster reaction rates and larger yields of nanoparticles but may also promote agglomeration and reduce the stability of the particles. The pH can also affect the rate of particle formation, with more acidic or alkaline conditions generally leading to faster reaction rates [62,89,90].

### 3.2. Factors Influencing the Shape and Size of Synthesized ZnONPs

The shape and size of synthesized ZnONP nanoparticles can be influenced by several factors, including the concentration of plant extracts and metal salts, pH, temperature and stirring rate. Higher concentrations of plant extracts and metal salts generally lead to larger particles, while more acidic or alkaline conditions may promote the formation of rod-shaped particles. pH is an important factor for the biosynthesis of ZnONPs and could alter the shape and size of the synthesized nanoparticles [39,91]. Higher temperatures and faster stirring rates can also promote the formation of smaller particles with more uniform shapes. However, other factors, such as the type of plant extract or microbial species and metal salt used, can also play a role in determining the final size and shape of the nanoparticles [47,92,93]. 

## 4. Characterization of Biosynthesized ZnONPs

The use of various analytical techniques such as UV-visible spectrophotometry, XRD, SEM, TEM, FTIR, DLS and zeta potential analyzer analysis in the characterization of biosynthesized ZnONPs has been extensively reported in the literature. These techniques provide valuable information on the physical and chemical properties of nanoparticles, including their size, shape, surface charge, crystallinity and surface functional groups. For instance, UV-visible spectrophotometry is commonly used to determine the optical properties of ZnONPs, including their absorption spectra and bandgap energy. In ZnO, like in any other semiconductor, there is a valence band (VB) and a conduction band (CB) separated by a bandgap of a few eV. The ZnO absorption peaks at the transitions between VB and CB. Under irradiation, when enough energy is provided, an electron can be promoted from VB to CB, which will be recorded by a spectrophotometer as an absorption band/peak. The energy value of this peak is related to the value of the bandgap. Recombination of the excited electron from CB with the hole from VB will produce the fluorescent emission at about 380 nm, which is called exciton recombination. The slight variation of the absorption peak appears due to the different intermediary electronic levels generated by impurities or lattice defects [44]. XRD analysis provides information on the crystalline structure and phase purity of nanoparticles. The crystal size of the biosynthesized ZnONPs is generally calculated on the basis of XRD analysis [44]. SEM and TEM techniques are used to visualize the morphology, size and shape of nanoparticles. In both TEM and DLS, the size of nanoparticles and particle size distribution can be determined. While in TEM, the shape and crystallinity can also be determined, in DLS, the obtained size is usually larger due the presence of a solvent layer on the nanoparticle surface. DLS and a zeta potential analyzer provide information on the particle size distribution and surface charge, respectively. 

**Figure 3:**
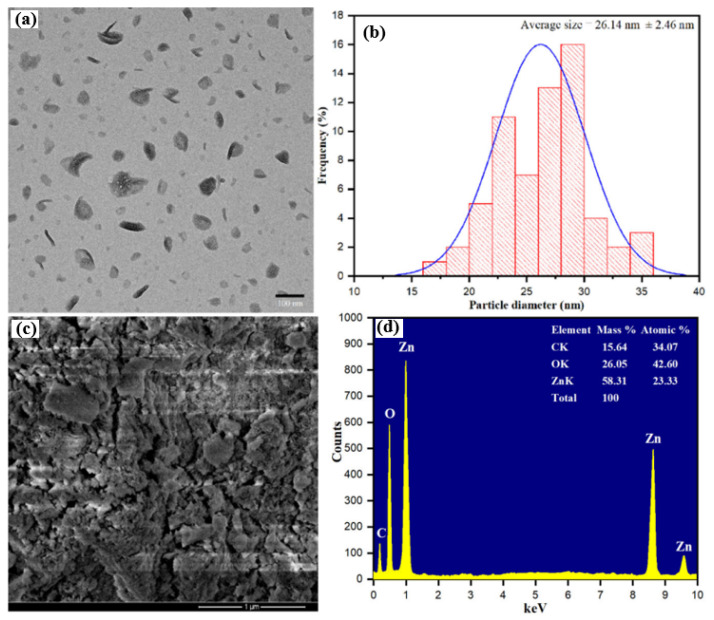
(**a**) Transmission electron microscope (TEM) scale bar: 100 nm; (**b**) particle size distribution histogram; (**c**) scanning electron microscopy (SEM) scale bar: 1 μm; (**d**) the EDX spectra of biosynthesized ZnONPs using leaf extract of *Salvia officinalis*. This figure has been reprinted with permission from Ref. [62], copyright 2021, MDPI.

FTIR analysis provides information on the functional groups present on the nanoparticle surface. The quantity of organics from plant or microbial extracts that are adsorbed on the ZnONP surface can be evaluated by thermal analysis. The chemical composition of produced ZnONP samples was also evaluated by using X-ray photoelectron spectroscopy (XPS) [36]. Several studies have reported the use of these techniques to characterize green-synthesized ZnONPs for various applications. For example, Faisal et al. [15] used UV-visible spectrophotometry, XRD, SEM, EDX and FTIR to characterize the *Paraclostridium benzoelyticum*-bacterium-mediated biosynthesized ZnONPs and investigate their antibacterial, antidiabetic, anti-inflammatory and antiarthritic activities. In another study, Supraja et al. [94] used FTIR, DLS and zeta potential analyzer analysis to characterize *Alstonia scholaris* stem-bark-extract-mediated ZnONPs and evaluate their antimicrobial efficacy. Abomuti et al. [62] used UV-visible spectrophotometry, Raman spectroscopy, SEM, TEM, XRD and FTIR to characterize the biosynthesized ZnONPs using leaf extract of *Salvia officinalis* and investigate their antimicrobial activity against *Candida albicans* isolates. TEM analysis revealed the wurtzite hexagonal shape of synthesized ZnONPs (Figure 3a), and the average size was 26.14 nm (Figure 3b). An SEM image revealed the aggregated form of synthesized ZnONPs and explored some rough, clumsy materials surrounding the ZnONPs (Figure 3c). EDX analysis confirmed the majority of ZnONPs present in the samples. Additional carbon peaks in the EDX spectrum suggested the presence of biomolecules such as vitamins, amino acids, polyphenols, flavonoids and saponins (Figure 3d). 

**Figure 4:**
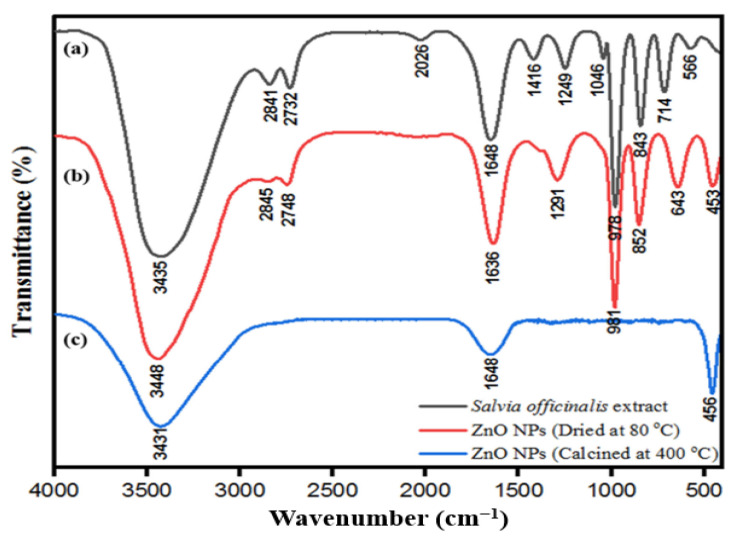
FTIR spectra of (**a**) aqueous leaf extract of *S. officinalis*, (**b**) ZnONPs dried at 80 °C, and (**c**) ZnONPs calcinated at 400 °C. This figure has been reprinted with permission from Ref. [62], copyright 2021, MDPI.

The FTIR spectrum also showed various biomolecules such as polyphenols and other biomolecules present in both aqueous leaf extract of *S. officinalis* and the synthesized ZnONPs, which suggested that these biomolecules are responsible for the synthesis and stabilization of ZnONPs and their biological activities (Figure 4a–c) [62].

Other studies have reported the use of these techniques to investigate the antimicrobial and antioxidant properties of green-synthesized ZnONPs for various applications. Sonia et al. [95] used UV-visible spectrophotometry, XRD, SEM and DLS to characterize biosynthesized ZnONPs and evaluate their antimicrobial and antioxidant potential for use in a cold-cream formulation. Barsainya and Singh [96] used XRD, SEM and TEM to characterize *Pseudomonas aeruginosa*-mediated ZnONPs and investigate their broad-spectrum antimicrobial effects. Overall, the use of various analytical techniques in the characterization of biosynthesized ZnONPs has provided valuable insights into their physical and chemical properties, enabling researchers to optimize their synthesis and tailor their properties for specific applications. Table 3 summarizes the different characterization techniques used for biosynthesized ZnONPs.

## 5. Antimicrobial Applications and Mechanisms of Biosynthesized ZnONPs

In recent years, there has been a growing interest in the development and utilization of nanomaterials for various applications, particularly in the field of antimicrobial research. Among these nanomaterials, ZnONPs have emerged as a promising candidate due to their unique physicochemical properties and potent antimicrobial activity. ZnONPs have been extensively studied for their ability to inhibit the growth of a wide range of microorganisms, including bacteria, fungi and viruses [24,42,62,99,100,101,102,103,104]. ZnONPs have potential applications in various fields, including food, agriculture, pharmaceuticals and biotechnology [43]. In the food and agriculture industries, ZnONPs have been shown to have potential applications as a food preservative and to enhance the antifungal activity of endophytic *Bacillus* sp. Fcl1. The extracts prepared from the *Bacillus* sp. Fcl1 cultured in the presence of ZnONPs had an increased production of lipopeptide surfactin derivatives and iturin, which are known for their antimicrobial properties [105]. In the medical field, ZnONPs have shown promise as antimicrobial agents for the treatment of various infections, including skin and wound infections, respiratory tract infections, and urinary tract infections. They have demonstrated broad-spectrum activity against both Gram-positive and Gram-negative bacteria, including multidrug-resistant strains. Furthermore, ZnONPs have been explored for their antifungal activity against pathogenic fungi, such as the *Candida* species, and have shown potential as antiviral agents against a range of viruses, including herpes simplex virus and influenza virus [106,107]. In the pharmaceutical industry, ZnONPs have been investigated for their potential use as a new antimicrobial agent to combat antibiotic-resistant bacteria. ZnONPs exhibited antimicrobial activity against methicillin-resistant *Staphylococcus aureus* (MRSA) and vancomycin-resistant *Enterococcus faecalis* (VRE) [108]. 

ZnONPs have also been investigated for their potential use in wound healing. The incorporation of ZnONPs into chitosan hydrogels improved their antimicrobial activity against *Staphylococcus aureus* and *Pseudomonas aeruginosa*. The use of ZnONPs in wound dressings could be a promising approach to preventing infections and promoting wound healing. In agriculture, ZnONPs have been utilized as antimicrobial agents for crop protection and disease management. They have been shown to effectively inhibit the growth of plant pathogens, including bacteria and fungi, offering an eco-friendly alternative to conventional pesticides. Additionally, the use of ZnONPs in food packaging materials has gained attention due to their antimicrobial properties, which can help extend the shelf life of perishable food products by inhibiting the growth of spoilage microorganisms. Moreover, ZnONPs have been investigated for their potential in environmental remediation, particularly in water treatment, where they can effectively eliminate waterborne pathogens and provide a sustainable approach for disinfection [40,109,110,111,112]. Studies have shown that the antimicrobial activity of ZnO nanoparticles is size-dependent, with smaller particles exhibiting higher antimicrobial activity due to their increased surface area and higher reactivity [113]. In addition, the shape of ZnONPs also plays a crucial role in their antimicrobial activity, with rod-shaped particles exhibiting higher activity than spherical particles [114].

**Figure 5:**
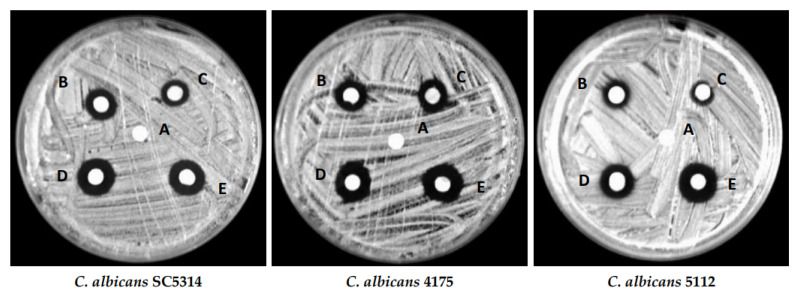
Zones of inhibition around discs impregnated with 1% DMSO (A), 2 µg/mL amphotericin B (B), ½ MIC of ZnONPs (C), MIC of ZnONPs (D), and MFC of ZnONPs (E) against different *Candida albicans* isolates. This figure has been reprinted with permission from Ref. [62], copyright 2021, MDPI.

The green synthesis approach utilizes plant extracts, microbes and waste biomaterials as reducing and stabilizing agents, thus reducing the use of hazardous chemicals and energy consumption during the synthesis process. Studies have shown that green synthesis methods produce ZnONPs with superior antimicrobial activity compared to those synthesized using chemical methods. For example, ZnONPs synthesized using aqueous extracts of *Heritiera fomes* and *Sonneratia apetala* mangrove plant species showed significant antimicrobial activity against *E. coli*, *S. aureus* and *B. subtilis* [92]. Similarly, *Alstonia scholaris* stem-bark-extract-mediated ZnONPs demonstrated significant antimicrobial activity against *P. aeruginosa*, *S. aureus* and *B. subtilis* [94]. There are many recent reports on the biosynthesis of ZnONPs using plants and microbes and their potential utilization to control drug-resistant pathogenic microorganisms (Table 1 and Table 2). Abomuti et al. [62] reported the biosynthesis of ZnONPs using leaf extract of *Salvia officinalis* and evaluated their antimicrobial activity against pathogenic *Candida albicans* isolates. They found that the biosynthesized ZnONPs strongly suppressed the growth of *C. albicans* isolates and showed a strong zone of inhibition (Figure 5). Faisal et al. [15] reported on the *Paraclostridium benzoelyticum*-bacterium-mediated extracellular synthesis of ZnONPs and evaluated their antimicrobial activity against *Helicobacter suis*, *H. felis*, *H. bizzozeronii* and *H. salomonis*. The biosynthesized ZnONPs strongly inhibited the growth of the tested pathogenic bacteria.

ZnONPs have gained significant attention as promising antimicrobial agents due to their unique physicochemical properties and broad-spectrum activity against various microorganisms. The antimicrobial mechanisms of ZnONPs involve a combination of physical, chemical and biological processes that collectively contribute to their efficacy in inhibiting the growth and survival of microorganisms [107,111,115]. One of the primary mechanisms by which ZnONPs exert their antimicrobial activity is through the generation of reactive oxygen species (ROS). ZnONPs can undergo redox reactions and produce ROS, such as superoxide radicals (O^2−^), hydrogen peroxide (H_2_O_2_) and hydroxyl radicals (OH·). These ROS are highly reactive and can cause oxidative stress in microbial cells by damaging cellular components, including lipids, proteins and nucleic acids. The accumulation of ROS disrupts normal cellular functions, leading to cell membrane damage, protein denaturation and DNA/RNA degradation, ultimately resulting in microbial cell death [111,116,117]. Moreover, ZnONPs possess a high surface-area-to-volume ratio, which enhances their contact with microbial cells and facilitates physical interactions. The small size of ZnONPs allows them to penetrate microbial cell membranes and enter the cytoplasm. Once inside the cell, ZnONPs can interact with intracellular components, such as enzymes and proteins, disrupting their structure and function. This disruption further contributes to the inhibition of microbial growth and proliferation [111,115,118]. Another important antimicrobial mechanism of ZnONPs is their ability to disrupt the integrity and permeability of microbial cell membranes. ZnONPs can interact with the lipid bilayer of the cell membrane, leading to membrane destabilization and increased membrane permeability. This disruption of the cell membrane integrity compromises the structural integrity of microorganisms and leads to leakage of cellular contents, loss of vital ions and ultimately cell death [111,115,119]. Furthermore, ZnONPs have been found to interfere with microbial enzyme activity. Certain enzymes, such as ATPases and respiratory chain enzymes, are crucial for microbial metabolism and energy production. ZnONPs can inhibit the activity of these enzymes, disrupting the energy balance and metabolic processes of microorganisms. This interference with enzyme activity further contributes to the antimicrobial effects of ZnONPs [111,120]. Table 4 summarizes the modes of action of biosynthesized ZnONPs against different pathogenic microbes.

**Figure 6:**
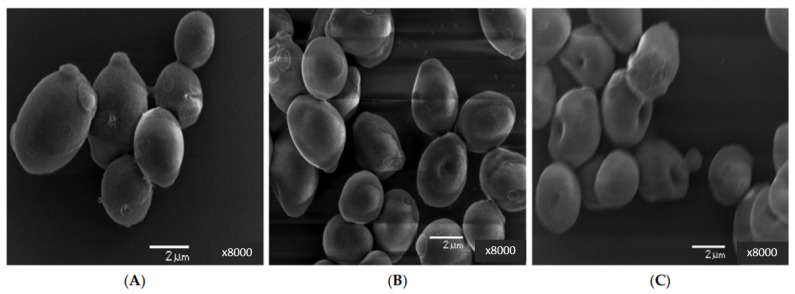
Scanning electron micrographs (SEM) of *C. albicans* SC5314: (**A**) represents untreated control cells, whereas (**B**,**C**) represent the cells exposed to MIC and MFC of biosynthesized ZnONPs, respectively. This figure has been reprinted with permission from Ref. [62], copyright 2021, MDPI.

Abomuti et al. [62] applied plant-mediated biosynthesized ZnONPs to treat the pathogenic *C. albicans* isolates and found that the biosynthesized ZnONPs damaged the cell wall and cell membrane of *C. albicans* and inhibited the production of ergosterol, which lead to the death of the cell (Figure 6). 

**Figure 7:**
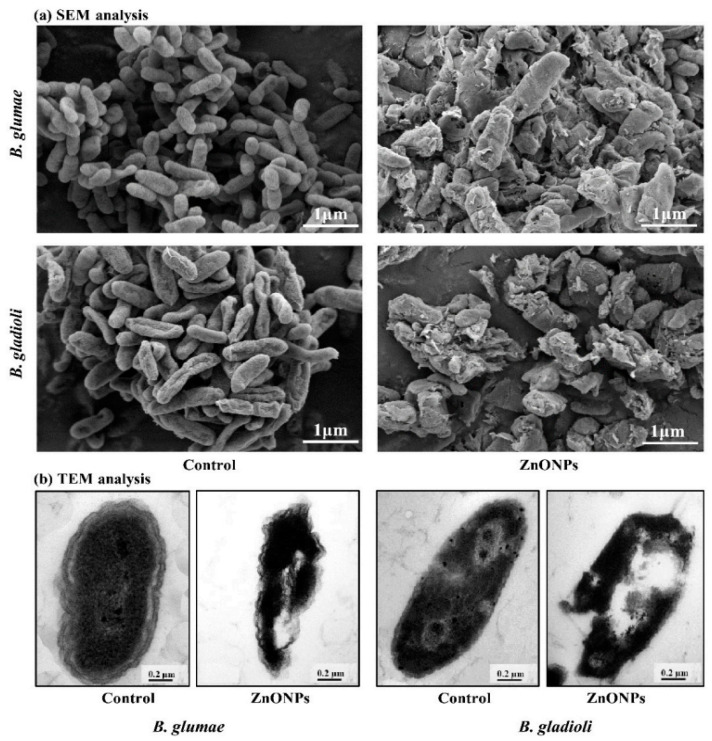
SEM (**a**) and TEM (**b**) images of rice bacterial pathogen *B. glumae* and *B. gladioli* cells after 8 h treatment with (50 µg mL^−1^) and without (control) biogenic ZnONPs. This figure has been reprinted with permission from Ref. [42], copyright 2021, MDPI.

According to Ahmed et al. [42], bacterial-mediated biosynthesized ZnONPs effectively control the growth of pathogenic microorganisms *B. glumae* and *B. gladioli*. They reported that synthesized ZnONPs damaged the cell membrane, proteins, ribosome and cytoplasmic materials of *B. glumae* and *B. gladioli*, produced reactive oxygen species and were involved in the leakage of genetic materials, resulting in cell death (Figure 7).

It is important to note that the antimicrobial mechanisms of ZnONPs can vary depending on the type of microorganism and the specific conditions. While ZnONPs exhibit broad-spectrum antimicrobial activity, some microorganisms may exhibit varying degrees of susceptibility due to differences in cell wall composition, membrane structure or defense mechanisms. In conclusion, ZnONPs possess multiple antimicrobial mechanisms that collectively contribute to their effectiveness in inhibiting the growth and survival of microorganisms. These mechanisms include the generation of reactive oxygen species, physical interactions, disruption of cell walls and cell membranes, damage of DNA, interference with microbial enzyme activity, protein denaturation, ribosomal destabilization and mitochondrial dysfunction. Understanding the antimicrobial mechanisms of ZnONPs is crucial for the development of novel antimicrobial strategies and the optimization of their application in various fields, including medicine, food industry, agriculture and environmental remediation.

## 6. Conclusions and Future Prospects

The use of green synthesis methods for the production of ZnONPs has emerged as a promising approach to achieve enhanced antimicrobial activity with reduced environmental impact. Biosynthesis of ZnONPs using microbes and plants is a facile, non-toxic, cost-effective and eco-friendly method. In this review, the biosynthesis of ZnONPs using microbes and plants has been comprehensively reviewed. The antimicrobial applications and mechanisms of the biosynthesized ZnONPs against various pathogenic microorganisms have also been highlighted. Plant extracts, microbial biomass or culture supernatant contain various biomolecules including enzymes, amino acids, proteins, vitamins, alkaloids, flavonoids, etc., which serve as reducing, capping and stabilizing agents during the safe, facile and rapid biosynthesis of ZnONPs. The antimicrobial activity of ZnONPs is attributed to several mechanisms, including physical damage to microbial cell walls and cell membranes, production of reactive oxygen species and inhibition of microbial enzyme activity. As ZnONPs are edible and safe for utilization, ZnONPs could be potentially utilized in different food industries to control foodborne pathogens, as well as in many other sectors such as health care and agriculture to effectively control different pathogenic microorganisms. ZnONPs also exhibit antioxidant and wound-healing properties, making them suitable for use in cosmetics and dermatological formulations. 

In conclusion, the antimicrobial applications of ZnONPs hold great promise for the development of new antimicrobial agents to combat the growing threat of antimicrobial resistance. ZnONPs have potential applications in various fields, including food, agriculture, pharmaceuticals and biotechnology. It is worth mentioning that the safety and potential toxicity of ZnONPs are important considerations for their practical applications. While ZnONPs have demonstrated significant antimicrobial activity, their potential adverse effects on human health and the environment should be thoroughly evaluated. Proper characterization of ZnONPs, including size, shape and surface modifications, is crucial for understanding their interactions with biological systems and optimizing their antimicrobial efficacy while minimizing potential toxic effects.

## Figures and Tables

**Table 1:** Microbe-mediated biosynthesis and potential antimicrobial applications of ZnONPs. NA, Not Available.

Microbes Used for Synthesis	Synthesis Method	Optimum Synthesis Conditions (Salt Concentration, Temperature, Incubation Time)	Size (nm, Nanoparticles/Crystallite)	Shape	Target Pathogens	Reference
*Paraclostridium benzoelyticum*	Extra cellular	0.1 M zinc nitrate, 80 °C for 24 h	50 (Average)	Spherical and rectangular	*Helicobacter suis*, *H. felis*, *H. bizzozeronii*, *H. salomonis*	[15]
*Aspergillus* sp.	Extra cellular	0.1 N zinc acetate, 40 °C for 6 h	80–100	Sphere shape	*Escherchia coli*, *Pseudomonas aeruginosa*, *Salmonella typhi*	[3]
*Pseudomonas aeruginosa*	Extra cellular	2 mM zinc acetate, 35 ± 2 °C for 24 h	14.9 ± 3.5	Spherical	*Staphylococcus aureus*, *Escherichia coli*, *Bacillus subtilis*, *Pseudomonas aeruginosa*, *Candida albicans*	[44]
*Lactobacillus* spp.	Intracellular	500 mM zinc salt, 37 °C for 24 h	32 (Average)	Spherical	*Clostridium difficile*, *E. coli*, *Clostridium perfringens*, *S. typhi*, *Aspergillus flavus*, *C. albicans*	[8]
*Marinobacter* sp. 2C8 and *Vibrio* sp. VLA	Extra cellular	0.1 M zinc sulfate, 30 °C for 24 h	10.2–20.3	Spherical	*E. coli*, *P. aeruginosa*, *Listeria innocua*, *S. aureus*, *Bacillus subtilis*	[35]
*Bacillus cereus* RNT6	Extra cellular	0.1 zinc sulfate, 80 °C for 15 min	21–35	Spherical	*Burkholderia glumae*, *B. gladioli*	[42]
*Lactobacillus plantarum* TA4	Extra and intracellular	500 mM zinc salt, 24 h at 37 °C	152.8–613.5	Flower pattern	*E. coli*, *Salmonella* sp., *S. aureus*, *S. epidermidis*	[13]
Endophytic fungus *Alternaria tenuissima*	Extra cellular	2 mM zinc sulphate, at room temperature for 20 min	10–30	Spherical	*P. aeruginosa*, *Klebsiella pneumoniae*, *E. coli*, *S. aureus*	[24]
*Pseudomonas putida*	Combine of intra and extracellular	100 mg zinc nitrate into 100 mL culture solution, 24 h at 37 °C	44.5 (Average)	Spherical	*Pseudomonas otitidis*, *Enterococcus faecalis*, *Acinetobacter baumannii*, *P. oleovorans*, *B. cereus*	[45]
*Aeromonas hydrophila*	Intracellular	Zinc salt, 37 °C, for 24 h	57.7 (Average)	Spherical	*P. aeruginosa*, *Aspergillus flavus*	[46]
*Bacillus megaterium*	Intracellular	Zinc nitrate solution, 37 °C for 48 h	45–95	Rod and cubic	*Helicobacter pylori*	[47]
*Halomonas elongate*	Extracellular	Zinc chloride, 37 °C for one week	18.1 ± 8.9	Multiform	*E. coli*, *S. aureus*	[48]
*Lactobacillus paracasei* LB3	Intracellular	Zinc nitrate solution, 37 °C for 24 h	1179 ± 137	Spherical	*S. aureus*, *Acetinobacter baumannii*	[49]
*Lactobacillus sporogens*	Extracellular	0.1 M zinc sulfate, 37 °C for 24 h	145.7 (Average)	Hexagonal	*S. aureus*	[50]
*Rhodococcus pyridinivorans* NT2b	Extracellular	0.1 M zinc sulfate, 30 °C for 72 h	100–120	Roughly spherical	*S. epidermidis*	[51]
*Sphingobacterium thalpophilum*	Extracellular	Zinc nitrate solution, 37 °C for 24 h	40 (Average)	Triangle	*P. aeruginosa*, *Enterobacter aerogens*	[52]
*Staphylococcus aureus*	Extracellular	zinc acetate solution (1 mM), 37 °C.	10–50	Acicular	*S. aureus*	[53]
*Streptomyces* sp.	Extracellular	Zinc chloride solution, 28 °C for 7 days	20–50	Spherical	*E. coli*, *B. subtilis*	[54]
*Pichia kudriavzevii*	Extracellular	zinc acetate solution, 35 °C for 36 h	10–61	Hexagonal wurtzite	*B. subtilis*, *S. epidermidis*, *S. aurous*, *E. coli*, *Serratia marcescens*	[55]
*Pichia fermentas* JA2	Extracellular	1 mM zinc nitrate, 28 °C for 96 h	NA	Smooth and elongated	*P. aeruginosa*	[56]
*Aspergillus fumigatus* JCF	Extracellular	1.0 mM zinc sulfate, 32 °C for 96 h	60–80	Spherical	*K. pneumoniae*, *P. aeruginosa*, *E. coli*, *S. aureus*, *B. subtilis*	[57]
*Aspergillus niger*	Extracellular	5 mM Zinc nitrate, 32 °C for 48 h	61 ± 0.65	Spherical	*E. coli*, *S. aureus*	[58]
*Aspergillus terreus*	Extracellular	Zinc salt solution, 32 °C for 4 days	54.8–82.6	Spherical	*A. niger*, *A. fumigatus*, *A. aculeatus*	[59]

**Table 2:** Plant-mediated biosynthesis and potential antimicrobial applications of ZnONPs.

Plant	Used Part	Optimum Synthesis Conditions (Salt Concentration, Temperature, Incubation Time)	Size (nm, Nanoparticles/Crystallite)	Shape	Target Pathogens	Reference
*Punica granatum*	Peel extract	5 mM Zinc acetate, room temperature for overnight	10–45	Spherical	*Staphylococcus aureus*, *Bacillus subtilis*, *Pseudomonas aeruginosa*, *Escherichia coli*, *Candida albicans*	[63]
*Cassia siamea*	Leaf extract	1.0 mM zinc nitrate, heated for 3 to 4 h	13 (Average)	Spherical, oval, spheroidal	*Pseudomonas aeruginosa*, *Chromobacterium violaceum*	[14]
Cinnamon and bay	Leaves	Zinc salt, room temperature for 24 h	~10, 18.5 and ~30 (Average)	Spherical	*Staphylococcus aureus*, *Staphylococcus epidermidis*, *Escherichia coli*, *Klebsiella pneumoniae*	[9]
*Allium sativum*, *Zingiber officinale*	Bulb extract, root extract	Zinc acetate solution, 50 °C for 2 h	19.8, 21.9 and 23.9 (Average)	Wurtzite	*Escherichia coli*, *Pseudomonas putida*, *Staphylococcus aureus*, *Streptococcus pyogenes*	[2]
*Pisonia Alba*	Leaf extract	0.1 M zinc acetate, 70 °C for 2 h	Aggregated	NA	*Staphylococcus aureus*, *Klebsiella pneumoniae*	[36]
*Sargassum muticum*	Plant extract	5 mM zinc nitrate, 70 °C for 20 min and room temperature for 2 h	15–50	Wurtzite hexagonal	*Bacillus flexus*, *Bacillus filamentosus*, *Acinetobacter baumannii*, *Pseudomonas stutzeri*	[68]
*Punica granatum* peel and coffee ground	Plant extract	10 mM zinc acetate, 1 h at 70 °C	118.6, 115.7 and 111.2 (Average)	Nanorod	*Pseudomonas aeruginosa*, *Staphylococcus aureus*, *Klebsiella pneumoniae*, *Enterobacter aerogenes*	[69]
*Myrica esculenta*	Fruits extract	0.5 M zinc acetate, 40 °C for 2 h	31.7 (Average)	NA	*Fusarium oxysporum*, *Staphylococcus aureus*, *Pseudomonas aeruginosa*, *Rosellinia necatrix*, *Escherichia coli*	[23]
Gardenia thailandica triveng	Leaves	Zinc acetate solution, 70 °C for 30 min, room temperature for 1 h	37.4 (Average)	Spherical	*Pseudomonas aeruginosa* clinical isolates	[64]
*Cocos nucifera*	Extract	1 M zinc nitrate, 4 h at ambient temperature	28–59	Rock shaped	*S. aureus*, *E. coli*, *B. subtilis*, *K. pneumoniae*	[70]
*Clitoria ternatea*	Flower extract	0.1 M zinc nitrate, 4 h at 80 °C	40–81	Rod	*S. aureus*, *E. coli*	[71]
*Carica papaya*	Leaf extract	0.1 M Zinc acetate, 4 h at 80 °C	15–50	Semi-spherical	*Rosellinia necatrix*, *Sclerotinia sclerotiorum*, *Fusarium* spp.	[65]
*Tagetes erecta*	Flower extract	1.5 mM zinc nitrate, 24 h at 60 °C	30–50	Spherical	*E. coli*, *S. aureus*	[72]
*Spinacea oleracea*	Extract	Aqueous zinc acetate solution, 24 h at 60 °C	13.0 (Average)	granular	*Pseudomonas aeruginosa*	[73]
*Salvia officinalis*	Leaf extract	0.1 M zinc nitrate, 4 h at 50 °C	26.1 (Average)	Wurtzite hexagonal	*Candida albicans* isolates	[62]
Orange	Peel extract	1 M zinc nitrate, 2 h at room temperature	20–60	cubic	*Pseudomonas aeruginosa*, *B. subtilis*	[66]
*Phoenix dactylifera*	Waste	5 g zinc nitrate in 50 mL of extract, 30 min at room temperature	30 (Average)	Spherical	*Streptococcus pyogenes*, *Pseudomonas aeruginosa*, *Staphylococcus aureus*	[38]
*Brassica rapa*	Leaf extract	Zinc nitrate solution, 4 h at 80 °C	27.5 (Average)	Irregular	*Micrococcus luteus*, *Enterobacter aerogenes*	[74]
Red Paprika	Aqueous plant extract	2 M Zinc acetate, 6 h at room temperature	70–80	Rod	*S. enterica*.	[75]
*Aloe barbadense*	Leaf extract	10 mM zinc nitrate, 60 °C	44 (Average)	Quasi-hexagonal	*Bacillus subtilis*, *Bacillus licheniformis*, *Klebsiella pneumonia*, *Escherichia coli*, *Candida albicans*, *Aspergillus niger*	[76]
*Geranium robertianum*	Flower extract	10 mM zinc acetate, 2 h at room temperature	40 (Average)	Irregular	*Escherichia coli*, *Pseudomonas aeruginosa*, *Acinetobacter baumannii*, *Staphylococcus* *aureus isolates*	[67]
*Ocimum americanum*	Plant extract	1 mM zinc nitrate, 1 h at 60 °C	21 (Average)	Spherical	*B. cereus*, *Staphylococcus aureus*, *Klebsiella pneumonia*, *Vibrio parahaemolyticus*, *Pseudomonas aeruginosa*, *Escherichia coli*, *Salmonella typhi*, *Candida albicans*, *Xanthomonas citri*, *Aspergillus parasiticus*	[77]
*Azadirachta indica*	Leaves	Zinc nitrate solution, boiled at 350 ± 10 °C for 4 min	9–38	Hexagonal	*Klebsiella aerogenes* and *Staphylococcus aureus*	[78]
*Cannabis sativa*	Leaf	Zinc acetate solution, 80 °C for 12 h	34–38	Spherical	*Escherichia coli*, *Klebsiella pneumonia*, *MRSA*, *Pseudomonas aeruginosa*, *Salmonella typhi*, *Staphylococcus aureus*	[79]
*Carica papaya*	Latex	Zinc nitrate solution, 37 °C for 36 h	11–26	Hexagonal	*Pseudomonas aeruginosa* and *Staphylococcus aureus* compared to *Klebsiella aerogenes* and *Pseudomonas desmolyticum*	[80]
*Dolichos lablab* L.	Leaf	Zinc acetate solution, incubated 70 °C for 1 h	29 (Average)	Hexagonal	*Bacillus pumilus* and *Sphingomonas paucimobilis*	[81]
*Tabernaemontana* *divaricata*	Green leaf	Zinc nitrate solution, 80 °C until precipitation.	20–50	Spherical	*Salmonella paratyphi*, *Escherichia coli* and *Staphylococcus aureus*	[41]
*Moringa oleifera* (drumstick)	Leaves	Zinc acetate solution, 24 °C for 1 h	52 (Average)	Hexagonal wurtzite	*Bacillus subtilis* and *Escherichia coli*	[82]
*Mussaenda frondosa*	Leaf/stem	Zinc nitrate solution, 400 °C for 10–30 min	5–20	Spherical	*Staphylococcus aureus* and *Bacillus subtilis*	[83]
*Phyllanthus emblica*	Plant extract	Zinc chloride solution, 90 °C for 2 h	Aggregated	square-shaped	*S. pyogenes*, *S. aureus*, *S. typhi* and *E. coli*	[84]
*Plectranthus amboinicus*	Plant extract	Zinc sulfate solution, room temperature for 2 h	Aggregated	Irregular aggregated nanoflakes	*S. aureus* and *E. coli*	[85]

**Table 3:** Different characterization techniques used for biosynthesized ZnONPs.

Characterization Technique	Principle	Advantage	Reference
UV-visible spectrophotometry	Measures absorbance of light	Rapid and nondestructive	[97]
X-ray diffraction (XRD)	Measures crystal structure and size	Provides detailed crystallographic information	[62]
Scanning electron microscope (SEM)	Provides surface morphology and size	High resolution imaging	[98]
Transmission electron microscope (TEM)	Provides detailed information on size, shape and structure	High resolution imaging and analysis of individual particles	[62]
Fourier transform infrared spectroscopy (FTIR)	Measures functional groups on the nanoparticle surface	Provides information on surface chemistry	[97]
Dynamic light scattering (DLS)	Measures particle size distribution	Rapid and nondestructive	[97]
Zeta potential analyzer	Measures the surface charge of particles in solution	Provides information on particle stability	[97]

**Table 4:** Modes of action of green synthesized ZnONPs against pathogenic microbes.

Treated Pathogenic Microbes	Mode of Action	References
*H. suis*, *H. felis*, *H. bizzozeronii*, *H. salomonis*	Lead to the damage of cell wall, cell membrane and DNA, mitochondrial dysfunction, apoptosis, generation of reactive oxygen species and, finally, cell death.	[15]
*S. aureus*, *E. coli*, *B. subtilis*, *P. aeruginosa*, *C. albicans*.	Inhibit different metabolic functions including cell metabolisms, transportation, enzyme activity, etc.; generate reactive oxygen species and lead to the death of cell.	[44]
*S. aureus*, *E. coli*, *E. faecalis*, *S. enteritidis*, *K. pneumoniae*, *P. aeruginosa*, *A. baumannii*, *S. typhimurium*, *C. albicans*	Damage of cell membrane and DNA, leakage of intracellular molecules, denaturation of enzymes and proteins, inhibition of protein synthesis, generation of reactive oxygen species.	[121]
*Burkholderia glumae*, *B. gladioli*	Damage cell membrane, proteins, ribosome, and cytoplasmic materials; produce reactive oxygen species and cause leakage of genetic materials, resulting cell death.	[42]
*E. coli*, *Salmonella* sp., *S. aureus*, *S. epidermidis*	Damage the cell membrane, cause leakage of intracellular materials and generate reactive oxygen species, which lead to the death of the cell.	[13]
*S. aureus*, *K. pneumoniae*	Generation of reactive oxygen species, DNA damage, protein denaturation and mitochondrial dysfunction.	[36]
*P. aeruginosa*, *C. violaceum*	Attach to cell membrane, break membrane permeability, release Zn ions, generate reactive oxygen species.	[14]
*E. coli*, *P. putida*, *S. aureus*, *S. pyogenes*	Interact with cell membrane, produce reactive oxygen species, damage cell wall, DNA, protein and iron.	[2]
*B. flexus*, *B. filamentosus*, *A. baumannii*, *P. stutzeri*	Damage of cell wall, inhibition of cellular metabolism and respiration, destruction of DNA and inactivation of protein.	[68]
*C. albicans*	Disrupt and deform the cell wall and cell membrane and inhibit the production of ergosterol, which lead to cell death.	[62]
*S. pyogenes*, *P. aeruginosa*, *S. aureus*	Production of significant oxygen reactive species including hydroxyl radicals, superoxides and hydrogen peroxide.	[38]
*F. oxysporum*, *S. aureus*, *P. aeruginosa*, *R. necatrix*, *E. coli*	Damage cell membrane, generate reactive oxygen species, damage DNA, denature protein, cause ribosomal destabilization and mitochondrial dysfunction, which lead to the death of cell.	[23]

## Data Availability

Not applicable.
